# *In vitro* evaluation of 3D-printed conductive chitosan–polyaniline scaffolds with exosome release for enhanced angiogenesis and cardiomyocyte protection†

**DOI:** 10.1039/d5ra02940f

**Published:** 2025-05-20

**Authors:** Amir Hashemi, Masoumeh Ezati, Inna Zumberg, Larisa Chmelíková, Zdenka Fohlerová, Valentýna Provazník

**Affiliations:** a Department of Biomedical Engineering, Faculty of Electrical Engineering and Communication, Brno University of Technology Technicka 3082/12 61600 Brno Czech Republic amir.hashemi@vut.cz masoumeh.ezati@vut.cz provaznik@vut.cz; b Department of Microelectronics, Faculty of Electrical Engineering and Communication, Brno University of Technology Technicka 3082/12 61600 Brno Czech Republic

## Abstract

Myocardial infarction (MI) often results in significant damage to heart tissues, leading to cardiac dysfunction, fibrosis, and diminished cell–cell communication. Exosomes (EXOs) from stem cells show great potential in promoting tissue repair and angiogenesis, but their rapid clearance and degradation *in vivo* limit therapeutic efficacy. Here, we introduce a 3D-printed *in vitro* scaffold using a conductive biomaterial ink composed of chitosan (CS) and polyaniline (PANI). This scaffold combines the bioactivity of EXOs with the conductive properties of PANI to protect cardiac cells under ischemic stress. Using an *in vitro* hypoxia/reoxygenation (H/R) model with HL-1 cardiomyocytes, we simulated key aspects of myocardial ischemia-reperfusion injury. The addition of PANI improved the electrical conductivity of the scaffold, which was essential for enhancing cardiomyocyte viability and intercellular connectivity under hypoxic conditions. EXOs significantly promoted angiogenic activity *in vitro*, as evidenced by enhanced human umbilical vein endothelial cell (HUVEC) migration and robust tube formation, highlighting their role in stimulating new blood vessel growth. Molecular analyses revealed that EXOs positively influence processes such as angiogenesis and inflammation regulation in HL-1 cells. Additionally, EXOs improved HUVEC migration, emphasizing their pro-angiogenic role. These findings indicate that combining PANI and EXOs in a 3D-printed scaffold yields synergistic benefits, improving cardiomyocyte function and promoting endothelial angiogenesis *in vitro*, thereby providing insights for future cardiac repair strategies.

## Abstract 2

Myocardial infarction (MI) often results in significant damage to heart tissues, leading to cardiac dysfunction, fibrosis, and diminished cell–cell communication.

## Graphical abstract

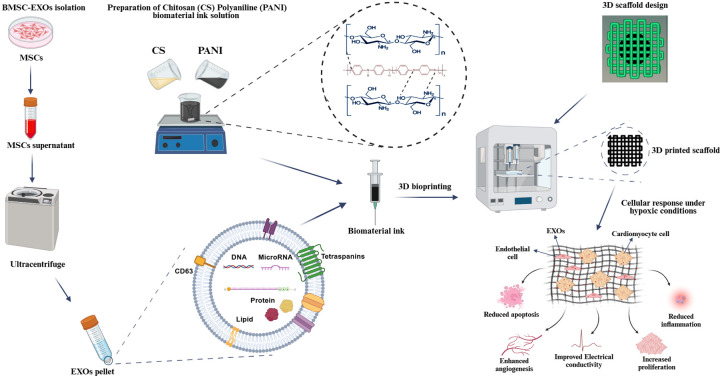

## 1. Introduction

MI continues to be a major cause of heart failure and death worldwide, mainly because of the obstruction of coronary arteries caused by the rupture of plaques in atherosclerosis. This obstruction hinders the delivery of nutrients to the heart muscle, resulting in ischemic injury and damage to the myocardium.^1^ Conventional treatments such as thrombolysis and percutaneous coronary intervention are designed to restore blood flow after a MI. Nevertheless, these interventions have the potential to exacerbate vascular endothelial cell dysfunction and disturb the balance of the cardiac microenvironment, leading to myocardial I/R injury and elevating the likelihood of heart failure.^2^ In the last twenty years, the integration of stem cell technology and biomaterials has brought about novel therapeutic approaches for restoring the heart after a MI. Mesenchymal stem cells (MSCs), specifically bone marrow MSCs (BMSCs), have attracted interest because of their ability to renew themselves, differentiate into multiple cell types, and exhibit strong signaling properties that affect nearby cells.^3^ The characteristics of BMSCs make them an auspicious choice for myocardial engineering. Nevertheless, injecting MSCs directly into the damaged myocardium has encountered obstacles such as inadequate cell attachment, limited cell retention, and low rates of cell survival, which have impeded their effectiveness in clinical applications. The majority of transplanted MSCs are swiftly eliminated from the heart, with only a minor portion persisting in the long run.^4^

EXOs, which are tiny extracellular vesicles released by cells, have become a potential substitute for directly transplanting stem cells. EXOs play a crucial role in facilitating communication between cells by transporting important molecules such as cell-specific proteins, lipids, and genetic materials like mRNAs and microRNAs (miRNAs) to specific target cells.^5^ Under both normal and pathological conditions, they can modulate the function and behavior of recipient cells. EXOs derived from stem cells possess cardioprotective properties that are comparable to those of the parent cells.^6^ Additionally, they offer advantages such as decreased immunogenicity, tumorigenicity, and ethical concerns.^7^ In addition, EXOs have the advantage of being simpler to prepare, sterilize, and store, which makes them a more cost-effective option compared to therapies based on cells.^8^ Nevertheless, the swift elimination and brief duration of action within the body restrict their therapeutic efficacy.^9^ Systemic administration frequently results in rapid clearance by phagocytes and subsequent accumulation in organs such as the liver, spleen, and lungs, which can lead to side effects and raise biosafety concerns. Additional investigation is required to determine the safety and effectiveness of EXOs for the treatment of cardiovascular disease.^10^

PANI and other conductive polymers exhibit potential due to their ability to conduct electricity, resistance to environmental degradation, and compatibility with living organisms.^11^ PANI can connect gaps in electrical conduction in fibrotic areas of the heart muscle, which helps to coordinate the contraction of the heart muscle and decrease irregular heart rhythms.^12^ CS, a polysaccharide obtained from the outer shells of crustaceans, is noteworthy because it is compatible with living organisms, can be broken down naturally, and can enhance the attachment and growth of cells.^13^ The cationic nature of the substance enables it to engage in electrostatic interactions with biomolecules, thereby improving the stability of the framework and facilitating the delivery of therapeutic agents.^14^ CS undergoes gelation in slightly acidic environments, making it suitable for producing injectable scaffolds that can adapt to the intricate structure of myocardial tissue.^15^

Hydrogels, which have a high-water content and properties similar to tissues, are becoming increasingly popular as carriers for therapeutic agents in cardiac repair.^16^ These materials offer mechanical reinforcement to the thinned ventricular wall and mitigate adverse remodeling after MI.^17^ Recent progress has resulted in the development of bioactive hydrogels that improve the ability to retain and enhance the therapeutic effectiveness of cells and EXOs that are included within them. An example of this is a clinical trial which demonstrated that the intramyocardial injection of human umbilical cord mesenchymal stem cells (hUC-MSCs)-laden collagen hydrogel resulted in a significant decrease in the size of the damaged heart tissue in patients with ischemic heart disease, when compared to the use of hUC-MSCs alone.^18^ Similarly, another study reported that the delivery of mesenchymal stem cell-derived exosomes using a conductive hydrogel significantly improved cardiac function and reduced infarct size in a rat model of myocardial infarction.^19^ Recent studies have also demonstrated that combining EXOs therapy with electrically conductive biomaterials can enhance cardiac repair by supporting electrical coupling and paracrine signaling, further reinforcing the synergistic potential of this approach.^20–23^ Despite these advancements, there remains a critical need for bioactive hydrogels that not only support cell viability and functionality but also address the complex physiological environment of the damaged myocardium.

To tackle these difficulties, here, we have developed a 3D-printed *in vitro* scaffold using a conductive CS/PANI biomaterial ink. This scaffold was engineered to mitigate the effects of I/R injury by leveraging the synergistic effects of PANI and EXOs. PANI's conductive properties help facilitate electrical and mechanical integration, improving cell viability and reducing apoptosis under hypoxic conditions. EXOs deliver bioactive molecules that can reduce inflammatory cytokine expression and stimulate angiogenesis. Herein, we evaluate the combined effects of this conductive scaffold with sustained EXO release using an *in vitro* H/R injury model with HL-1 cardiomyocytes, along with assays of endothelial angiogenesis, to provide insight into their therapeutic potential for myocardial repair. We focus on demonstrating enhanced angiogenic activity and cardiomyocyte protection *in vitro*, as a foundational step toward future *in vivo* applications.

## 2. Materials and methods

### 2.1. Materials

Aniline, chitosan (medium molecular weight), agarose, fetal bovine serum (FBS), acetic acid, sodium hydroxide (NaOH), phosphate buffered saline (PBS), dimethyl sulfoxide (DMSO) penicillin/streptomycin (PS), acetone, hydrochloric acid (HCl), DAPI (4′,6-diamidin-2-fenylindol), ammonium persulfate (APS), uranyl acetate, paraformaldehyde, norepinephrine, l-glutamine, Dulbecco's Modified Eagle Medium (DMEM), Claycomb medium and trypsin/ethylene diamine tetra-acetic acid (EDTA), DiI(1,1′-dioctadecyl-3,3,3′,3′-tetramethylindocarbocyanine perchlorate), HEPES (4-(2-hydroxyethyl)-1-piperazineethanesulfonic acid), Hank's Balanced Salt Solution (HBSS) and Thiazolyl Blue Tetrazolium Bromide were purchased from Sigma-Aldrich. Pierce™ BCA Protein Assay Kit, CD63 Monoclonal Antibody, HRP-conjugated secondary antibodies, cDNA Synthesis Kit, and ActinGreen 488 dye were obtained from Thermo Fisher Scientific. ECGM-MG endothelial cell growth medium was obtained from Cell Lines Service (CLS), RNeasy Mini Kit was purchased from Qiagen, (Hilden, Germany), and LightCycler 480 High Resolution Melting Master SYBR Green amplification kit was purchased from Roche (Basel, Switzerland).

### 2.2. Cell culture

The mice-derived HL-1 cardiomyocytes were acquired from Sigma-Aldrich. The cells were cultured in Claycomb medium supplemented with 10% FBS, 1% PS, 1% norepinephrine, and 1% l-glutamine, following the protocol outlined in the literature.^24^ BMSCs were cultured in DMEM containing 10% FBS and 1% PS. The cells were maintained at a temperature of 37 °C in a humidified atmosphere with 5% CO_2_. To ensure optimal growth and viability, the culture media for all cell types were replenished every two days. For the biological experiments, the cells were detached using trypsin, then mixed with fresh culture medium, and used in accordance with the experimental protocols. In addition, human umbilical vein endothelial cells (HUVECs) were purchased from CLS Cell Lines Service GmbH (Eppelheim, Germany) and cultured in ECGM-MG complete medium, which was supplemented with 1% PS.

### 2.3. Isolation and identification of BMSCs-EXOs

BMSCs were cultivated in flasks until they reached a cell density of 85%. Subsequently, the medium was replaced with DMEM containing 10% EXO-free FBS to ensure that the EXOs obtained originated solely from the BMSCs. To avoid contamination, the cell culture was performed using EXO-depleted FBS, as regular FBS contains elevated levels of EXOs. EXOs were extracted from the conditioned media following 48 h of incubation in a serum-free environment. Following centrifugation at 300*g* for 10 min to separate cellular debris, microvesicles were subsequently removed by centrifugation at 10 000*g* for 30 min. The media that had been treated was subsequently subjected to ultracentrifugation at a force of 100 000 times the acceleration due to gravity at a temperature of 4 °C for 120 min. The liquid portion was taken out, and the exosome solid was mixed again in a solution of 1× PBS and cleaned by spinning it at a very high speed of 100 000*g* at a temperature of 4 °C for an additional 90 min. The EXOs were subsequently re-suspended in 100 μL of 1× PBS and stored at −80 °C for future utilization.

The isolated EXOs were deposited onto transmission electron microscope (TEM) grids that were coated with formvar/carbon. The grids were then treated with glow discharge and stained with a 2% solution of uranyl acetate. Finally, the EXOs were examined using a TEM operating at 80 kV (Titan Themis 60–300 Cubed, Thermo Fisher Scientific, USA). The hydrodynamic size and morphology of the isolated EXOs were evaluated using dynamic light scattering (DLS) with a Zetasizer ZS90 instrument (Malvern Instruments Ltd, UK). Western blot analysis was used to confirm the presence of the exosomal marker protein CD63. CD63 was isolated using RIPA buffer and measured using a BCA Protein Assay Kit. The protein was separated using SDS-PAGE, followed by transfer onto nitrocellulose membrane. Subsequently, the membrane blocked with skim milk for 2 h and then incubated for another 2 h at room temperature with primary antibodies against CD63 protein, followed by 3 times washing with wash buffer. The membrane then incubated with HRP-conjugated secondary antibodies for 45 min at room temperature. At the final point, DAB substrate kit was used for the chromogenic detection of CD63 protein.

### 2.4. Cellular uptake of BMSCs-EXOs

BMSCs-EXOs were stained with 1 mM DiI (1,1′-dioctadecyl-3,3,3′,3′-tetramethylindocarbocyanine perchlorate) dye and subjected to a 5-min incubation at 37 °C to investigate the process of exosome uptake. A control group that did not receive EXOs was used as the negative control. A total of 4000 HL-1 cells were placed in 35 mm Glass bottom dishes with 14 mm well size (D35-14-1.5-N, Cellvis). The cells underwent a 24-h period of nutrient deprivation, followed by two rounds of rinsing with PBS. EXOs labeled with DiI were introduced, and the cells were incubated at a temperature of 37 °C and 5% CO_2_ for an additional 24 h. Following exposure, the cells were treated with a 4% paraformaldehyde solution for 20 min to ensure fixation. Subsequently, the specimens were treated with a green fluorescent dye specifically designed for F-actin, following the guidelines provided by the manufacturer. The nuclei were labeled with DAPI. Leica TCS SP8 confocal laser scanning microscope (Leica Microsystems, Germany) was used to visualize fluorescence signals.

### 2.5. Scratch migration assay

For migration tests, HUVECs were placed on a 35 mm glass bottom dish with 20 mm well size (D35-20-1.5-N, Cellvis) at a density of 3 × 10^5^ cells and kept at 37 °C with 5% CO_2_ to encourage cell attachment and the formation of a dense monolayer. After the cells had merged together, a clean 200 μL pipette tip was used to remove the cell layer gently. The medium was extracted, and the cells were rinsed with PBS. Subsequently, a fresh solution containing BMSCs-EXOs at concentrations of 50 μg mL^−1^ was introduced. The samples were examined at 24 h after treatment using a confocal microscope. The MATLAB software was used to analyze each dish in three separate areas based on a previous study,^25^ and each scratch test was conducted three times.

### 2.6. *In vitro* angiogenesis measurement

*In vitro* angiogenesis assays were performed using HUVECs. To begin, 50 μL of gel was added to a confocal dish and allowed to solidify for 45 min at 37 °C with 5% CO_2_. HUVECs, previously labeled with CellTracker Green CMFDA and at passage 2 or 3, were then seeded onto the gel at a density of 1 × 10^4^ cells per dish. After 24 h of incubation, tube formation was examined using a Leica DMi8 phase contrast microscope equipped with a CCD camera (LEICA DFC7000 T). Image analysis was carried out with ImageJ software, where tube length and width were measured, and the number of loops, defined as fully enclosed circular structures, was quantified.

### 2.7. Biomaterial inks preparation

#### 2.7.1. Synthesis of PANI

PANI was synthesized using a chemical oxidation polymerization method, following the procedure described by Stejskal *et al.*,^26^ with minor adjustments. A solution containing 9.1 mL of aniline in 250 mL of 1 M HCl was combined with a solution containing 28.55 g of APS 250 mL in deionized water. Following 15 min of agitation, the emergence of dark green solid particles was observed, indicating the initial formation of PANI in the emeraldine salt state. Subsequently, the solution was allowed to stand undisturbed at 15 °C for 24 h in order to enhance the production and molecular weight of PANI. The produced PANI was separated by filtration using Whatman filter paper (no. 42; pore size 2.5 μm). It was subsequently rinsed three times with a 0.2 M HCl solution, and then with acetone, in order to eliminate any remaining initiators, monomeric units, low molecular weight oligomers, and other impurities. Ultimately, the PANI powder underwent a drying process in an oven at 60 °C for 24 h.

#### 2.7.2. Preparation of CS hydrogel

In order to create the CS biomaterial ink hydrogel, 500 mg of CS was dissolved in 10 mL of a 1% acetic acid solution, resulting in the formation of a viscous mixture. The solution was agitated continuously at room temperature using a magnetic stirrer for 4 h to guarantee thorough dissolution and uniformity. To replicate physiological conditions and ensure biocompatibility, sodium bicarbonate was slowly introduced to the solution to adjust the pH to 7.2, considering its acidic nature. This control hydrogel was used to compare the effects of the conductive and bioactive components in the CS–PANI–EXOs formulation.

#### 2.7.3. Formulation of CS–PANI–EXOs biomaterial ink

To prepare the conductive biomaterial ink, 100 mg of the synthesized PANI was initially dispersed in 3 mL of deionized water, resulting in a homogeneous PANI solution. This solution was then introduced into the CS hydrogel in varying volumes: 250 μL, 500 μL, and 750 μL, respectively, for each formulation. The PANI solution was added slowly to the CS hydrogel solution while stirring continuously. The incorporation of PANI into the CS matrix was catalyzed by adding 30 μL of paraformaldehyde. This catalysis promotes cross-linking between PANI and CS, resulting in the formation of a stable CS–PANI composite hydrogel. The reaction was allowed to continue for 24 h at room temperature to ensure full cross-linking and even dispersion of PANI within the hydrogel matrix. A solution of isolated BMSCs-EXOs, with a concentration of 50 μg mL^−1^, was prepared and then added to the CS–PANI hydrogel. The mixture was gently stirred to guarantee a complete mixture and uniform dispersion of EXOs throughout the biomaterial ink.

### 2.8. Printability

The printability of the biomaterial inks was evaluated using the INKREDIBLE+ 3D printer (Cellink, Gothenburg, Sweden). The scaffolds were prepared using various ink formulations, each tailored with specific printing parameters to enhance printability and ensure structural integrity. The CS ink was printed using an extruder temperature of 25 °C, a print-head speed of 10 mm s^−1^, and a printing pressure of 100 kPa. For the composite inks, which included CS–PANI and CS–PANI–EXOs, the extruder temperature was maintained at 25 °C, but the print-head speed was reduced to 5 mm s^−1^ to ensure precise deposition of the more complex formulations. The printing pressure for these composite inks was raised to 150 kPa respectively in order to handle the increased thickness and to achieve the best possible flow and extrusion properties. The adjustments were required in order to preserve the structural integrity and mechanical characteristics of the printed scaffolds. The inks were deposited using printing needles with a diameter of 0.34 mm (22G). Each formulation resulted in the printing of 3D cubic scaffold structures with precise dimensions of 13 mm in length, 13 mm in width, and 1 mm in height. These structures acted as a frame of reference for subsequent examination and characterization. The 3D designs were generated utilizing Autodesk Fusion 360 software, while the necessary g-codes were produced using Cellink HeartWare software.

### 2.9. Biomaterial inks characterization

To evaluate the rheological characteristics of the prepared biomaterial inks, oscillatory frequency sweep tests were performed using a Discovery Hybrid Rheometer-2 (TA Instruments, USA), equipped with a 40 mm parallel plate geometry set at a 500 μm gap. The experiments were conducted at room temperature, replicating the thermal conditions commonly found during the ink extrusion process. These tests covered an angular frequency range from 0.628 rad s^−1^ to 125.704 rad s^−1^. The complex viscosity (Pa S), storage modulus (*G*′), and loss modulus (*G*′′) of the inks were measured to determine their response to oscillatory shear. The FTIR spectra were obtained using a Vertex 70v vacuum spectrometer (Bruker, Germany) that had an Attenuated Total Reflectance (ATR) module for spectroscopy. The spectra were obtained in the wavelength range of 4500 to 0 cm^−1^.

Electrochemical impedance spectroscopy (EIS) measurements were conducted at room temperature using an electrochemical workstation (Metrohm Autolab). The experimental arrangement comprised gold interdigitated electrodes (conductance constant: 9.450 μm.) placed in the bottom of homemade chamber. The chamber was filled with degassed biomaterial ink and properly closed to avoid hydrogel drying. EIS measurements were performed at the frequency range from 0.1 Hz to 500 kHz, using an alternating current (AC) amplitude of ±10 mV. The total impedance (*Z*) values obtained from the EIS measurements were used to calculate the electrical conductivity (*σ*) of the hydrogels using the formula (1).1
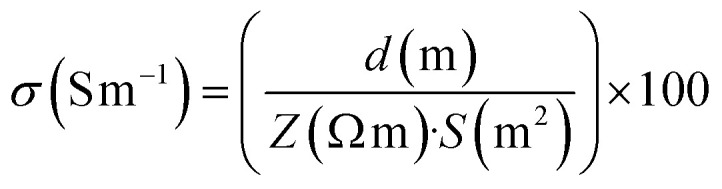
where *Z* is the total impedance at each frequency (normalized to the dimensions of the electrode setup), *S* is the surface area of the working electrode, and *d* is the distance between the fingers of the interdigitated electrodes. To estimate direct current (DC) conductivity, the real part of the impedance (*Z*′) at the lowest measured frequency (0.1 Hz) was used in eqn (1). This approach assumes that at low frequencies, capacitive effects are negligible and the response is dominated by resistance. The inter-electrode gap (*d*) was 9.450 μm and the effective surface area (*S*) was estimated at 1 mm^2^.

### 2.10. Identification and release profile of EXOs in biomaterial ink hydrogels

To visually illustrate the dispersion of EXOs within the hydrogel, the EXOs were labeled with the fluorescent dye DiI. The labeled EXOs were completely blended with 100 μL of the hydrogel and subsequently placed in a 35 mm Glass bottom dish with 20 mm well size (D35-20-1.5-N, Cellvis). The EXOs' distribution was examined through Leica TCS SP8 confocal laser scanning microscope and 3D images were obtained to ensure their even dispersion within the hydrogel matrix.

The *in vitro* evaluation of the EXO's release kinetics of CS–PANI–EXOs ink was conducted using a dynamic dialysis technique. A dialysis membrane with a molecular weight cutoff ranging from 8000 to 12 000 was used. 3 mL of the ink was placed inside a dialysis bag. The bag was subsequently submerged in a 4 mL solution of PBS at a pH of 6.5 and kept at 35 ± 0.5 °C. At regular intervals, the same volume of heated dissolution medium was added again after collecting a 1 mL sample. The released EXOs were then measured quantitatively using a micro BCA protein assay kit.

### 2.11. HL-1 cells viability on the 3D-printed scaffolds

An MTT assay was performed to evaluate the biocompatibility of different 3D-printed scaffolds. The scaffolds were sterilized using UV light for 20 min, and then were positioned in a 24-well plate. The proliferation studies involved seeding the samples with a concentration of 40 000 HL-1 cardiomyocyte cells per ml in each well. The samples were then incubated for 1, 3, and 5 days. Morphological observations were conducted using a Leica DMi8 phase contrast microscope equipped with a CCD camera (LEICA DFC7000 T). Following each incubation period, the medium was removed, and the samples were rinsed with PBS to eliminate any unattached cells. Subsequently, fresh DMEM media was introduced, along with 20 μL of MTT solution (5 mg mL^−1^ in fresh medium) to each well, resulting in a final volume of 200 μL. The samples were incubated for 3 h. Subsequently, the medium was extracted and DMSO was employed to dissolve the formed formazan crystals. The measurement of absorbance was conducted at a wavelength of 570 nm using a UV-vis spectrophotometer, with a background subtraction performed at 650 nm.

### 2.12. Observation of HL-1 cells beating on conductive and non-conductive scaffolds

The inherent rhythmic contractions of HL-1 cells grown on both conductive and non-conductive scaffolds were observed using a Leica DMi8 phase contrast microscope equipped with a CCD camera (LEICA DFC7000 T). Daily inspections were performed to observe the cellular activity in various regions of the cubic scaffolds. To ensure comprehensive coverage, each scaffold was meticulously examined in a minimum of six distinct regions, encompassing both the periphery and central areas. Particular emphasis was placed on identifying ‘center beating’, which refers to the occurrence of beating activity not only at the edges but also in the central regions of the scaffold. The video recordings were made to document the dynamic behavior of the cells on the scaffolds. Additional analysis was conducted using MATLAB, in which a 30-second video of the beating was converted into a series of 150 images (5 frames per second). The intensity of a specific spot in the center of each frame was measured to quantify the beating activity.

### 2.13. Calcium transient analysis

To examine calcium transients in HL-1 cells cultured on different scaffolds, the cells were initially seeded on the scaffolds and allowed to grow until they reached a confluency of approximately 95%. After reaching the desired level of cell growth, the culture medium was cautiously extracted, and the cells were gently rinsed with PBS to remove any remaining medium. A calcium-sensitive dye solution was created by mixing 1 μL of Pluronic F-127 (20% concentration), 20 μL of HEPES, and 979 μL of HBSS, resulting in a total volume of 1 mL. To this solution, 5 μL of Fluo-8 (2 mM), a dye used to visualize calcium activity within cells, was added. Following that, the Fluo-8 dye solution was applied to the cells, ensuring that the entire scaffold surface was fully covered. The cells were placed in an incubator at 37 °C for an hour to ensure sufficient uptake and even dispersion of the dye. After the incubation period, the calcium transients were observed using a confocal microscope, and Image sequences were captured. The calcium transients were analyzed using MATLAB. To evaluate the calcium transient of HL-1 cells, the fluorescence intensity of a consistent spot was measured in all images. To compensate for differences in dye concentration, the fluorescent signals were adjusted by normalizing the fluorescence (*F*) concerning the baseline fluorescence (*F*_0_). The normalization process yielded accurate data on the fluctuations in intracellular calcium ([Ca_2+_]_*i*_) transients from their baseline values (*F*/*F*_0_), thereby eliminating any discrepancies in fluorescence intensity caused by variations in dye volumes.

### 2.14. Immunocytochemistry

Immunocytochemistry was performed 10 days after seeding to evaluate the quality of the expression and location of the gap junction protein Cx43 in HL-1 cells adhered to scaffolds. The HL-1 cells were initially treated with a solution consisting of equal parts acetone and ethanol, in a ratio of 50 : 50, for 10 min at a −20 °C. The samples were subsequently treated with a 0.1% solution of Triton X-100 in PBS to make them permeable, and then blocked using a 3% solution of FBS in PBS. The specimens were subjected to incubation with a rabbit-anti-Cx43 antibody, which was diluted at a ratio of 1 : 200 in a blocking buffer, for 2 h at room temperature. Following the washing step, a secondary antibody called Alexa Fluor 488 goat-anti-rabbit (Invitrogen) was introduced. It was diluted 1 : 200 in a blocking buffer and left to incubate for an additional 2 h at room temperature. In order to determine the cell count per unit area, HL-1 cells were stained with DAPI at a dilution of 1 : 36 000 from the stock solution. The stained cells were then dehydrated and mounted in Prolong Gold (Life Technologies). Leica TCS SP8 confocal laser scanning microscope was used to capture images of the HL-1 cells on the film surfaces. In order to prevent any bias, an observer who was unaware of the details conducted the analysis. The images were renamed and arranged in a random order. The ImageJ was utilized for image analysis according to a previous investigation.^27^ The quantification process involved counting the number of cells exhibiting positive staining for Cx43 at the periphery in each image, as well as counting the cells lacking peripheral expression of Cx43. Quantification was performed on three images per film to determine the percentage of cells exhibiting peripheral Cx43 expression. The mean value obtained from these measurements was utilized for statistical analysis, with 4 films examined for each condition.

### 2.15. H/R exposure and gene expression profiling of HL-1 cells

For the hypoxia exposure experiments, HUVECs and HL-1 cells were separately cultured on the scaffolds and placed in a Stage Top Digital Gas Chamber (Okolab, Italy), connected to a Leica TCS SP8 confocal laser scanning microscope for time-lapse observation. The chamber was set to maintain a hypoxic environment by controlling the gas mixture to consist of 1% O_2_, 5% CO_2_, and 94% N_2_.^28^ The cells were subjected to this hypoxic condition for 24 h. Following this period, the cells were transferred to a normoxic incubator with 5% CO_2_ to reoxygenate for 4 h.

To evaluate the impact of H/R on gene expression, the expression levels of BAX, BCL-2, TNF-α, IL-6, and VEGF genes in HL-1 cells and NF-κB and VEGF genes in HUVECs were measured using quantitative PCR (qPCR) on day 10 post-treatment. RNA was extracted using the RNeasy Mini Kit, and its concentration was determined with a NanoPhotometer (IMPLEN, Munich, Germany). Complementary DNA (cDNA) was synthesized using the RevertAid First Strand cDNA Synthesis Kit. The gene expression levels were quantified using the LightCycler 480 High Resolution Melting Master SYBR Green amplification kit on the croBEE® RT-PCR System (GeneProof, Brno, Czech Republic). The following primers were used for HL-1 cells:

BAX: AGACAGGGGCCTTTTTGCTAC (forward), AATTCGCCGGAGACACTCG (reverse)

BCL-2: GCTACCGTCGTGACTTCGC (forward), CCCCACCGAACTCAAAGAA (reverse)

TNF-α: CCTCCCTCTCATCAGTTCTA (forward), ACTTGGTGGTTTGCTACGAC (reverse)

IL-6: CTTGGGACTGATGCTGGTGA (forward), TTGCCATTGCACAACTCTTTTC (reverse)

VEGF: GGAGATCCTTCGAGGAGCACTT (forward), GGCGATTTAGCAGCAGATATAAGAA (reverse). The following primers were used for HUVECs:

NF-κB: ACACCGTGTAAACCAAAGCC (forward), AGCCAGTGTTGTGATTGCT (reverse).

VEGF: CTCCACCATGCCAAGTGGTC (forward), CTCATCTCTCCTATGTGCTG (reverse).

The PCR protocol included an initial denaturation at 95 °C for 5 min, followed by 35 cycles of amplification (95 °C for 30 seconds, 52 °C for 30 seconds, and 72 °C for 30 seconds). GAPDH served as the housekeeping gene, with the forward primer sequence GGTCGGAGTCAACGGATTTG and the reverse primer sequence ATGAGCCCCAGCCTTCTCCAT. Relative gene expression levels were calculated using the ΔΔCt method, with three biological replicates (*n* = 3) for each treatment group. Additionally, the expression level of CX-43 was analyzed using qPCR with the same protocol, except the annealing temperature was adjusted to 55 °C. The primers for the CX-43 gene were TCTGCTATGACAAGTCCTTCC (forward) and GCTTCTCTTCCTTTCTCATCAC (reverse).

### 2.16. Statistical analysis

The experiments were conducted in triplicate, and results are presented as mean ± standard error of the mean. Group comparisons were performed using one-way analysis of variance (ANOVA), with statistical significance set at *p < 0.05.

## 3. Results

### 3.1. Characterization of EXOs

**Fig. 1:**
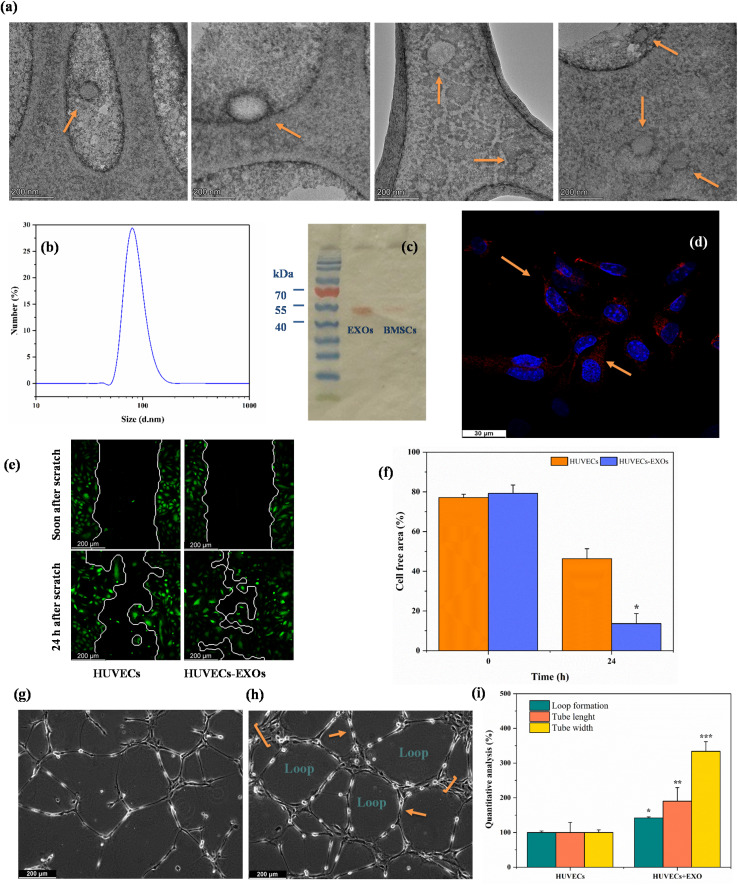
(a) Representative TEM images of isolated EXOs, exhibiting characteristic round morphology; orange arrows indicate individual EXOs. (b) DLS analysis indicating the size distribution of the EXOs, ranging from 50 to 150 nm. (c) Western blot analysis confirming the presence of the exosomal marker protein CD63 in the isolated EXOs. (d) Fluorescence microscopy image of DiI-labeled EXOs (red) introduced to HL-1 cells, showing predominant localization in the cytoplasm and some in the nuclear region (nuclei stained with DAPI in blue). The yellow arrows indicate the regions where EXOs are predominantly localized within the cells. (e) Scratch migration assay results showing HUVECs migration soon after scratch and 24 h post-scratch in control and EXOs-treated groups. (f) Quantitative analysis of cell-free area reduction in the scratch assay, illustrating significantly enhanced migration in the EXO-treated group compared to the control group after 24 h (*p < 0.05). (g and h) Phase-contrast microscope images from the tube formation assay illustrate the morphological differences between HUVECs cultured without (g) and with EXOs (h) treatment. Arrows highlight the newly formed tubular structures, while the label “Loop” identifies fully enclosed circular formations within the network. (i) Quantitative analysis of loop number, total tube length, and average tube width revealed that EXO treatment significantly enhanced all parameters assessed, confirming the strong pro-angiogenic activity of EXOs (*p < 0.05, **p < 0.01, ***p < 0.001).

The isolated BMSCs-EXOs were verified to possess the distinctive bilayer membrane structure and an average diameter of approximately 100 nm through TEM analysis (Fig. 1a). The findings were corroborated by the application of DLS analysis (Fig. 1b), which revealed that the EXOs exhibited a size distribution ranging from 50 to 150 nm, aligning with the standard dimensions of EXOs. A western blot analysis was performed to confirm the exosomal nature of the isolated particles. The results showed a distinct band at 53 kDa molecular weight, indicating the presence of the exosomal marker protein CD63 (Fig. 1c).

To facilitate tracking and visualization, the EXOs were labeled with the fluorescent dye DiI (Fig. 1d). Fluorescence microscopy revealed that, upon introduction to HL-1 cells, the DiI-labeled EXOs predominantly localized in the cytoplasm, with a lesser quantity observed in the nuclear region. This suggests that the EXOs were successfully taken up by the cells, which could potentially impact the cells' internal signaling and functions.

### 3.2. Scratch migration assay

The impact of EXOs on the migration and morphology of HUVECs was assessed through a scratch wound healing assay and the results are presented in Fig. 1e. Observations were conducted immediately following the creation of the scratch and then repeated after 24 h. The results demonstrated a significant enhancement in cell migration in the group that received treatment with EXOs, as compared to the untreated control group. After exactly 24 h from the scratch, the HUVECs treated with EXOs had almost completely recovered the scratch, resulting in a significant reduction in the area without cells. On the other hand, the control group was only able to close 62% of the area without cells. The observed disparity was statistically significant (*p < 0.05), suggesting that the administration of EXOs substantially improves the motility and migration of HUVEC. Fig. 1f illustrates these results, demonstrating a significantly reduced cell-free area in the group treated with EXOs compared to the control group after 24 h. This result demonstrates the capacity of EXOs to enhance the movement of endothelial cells, a critical factor in activities such as wound healing and the restoration of blood vessels.

### 3.3. *In vitro* angiogenesis

To assess the pro-angiogenic potential of EXOs, a tube formation assay was conducted using HUVECs (Fig. 1g–i). In the absence of EXOs, HUVECs generated limited and discontinuous capillary-like structures (Fig. 1g). In contrast, BMSC-EXO treatment markedly enhanced tube formation, resulting in a denser and more organized vascular network within 24 h (Fig. 1h). EXOs-treated cultures displayed numerous branch points and enclosed loops, indicative of advanced network complexity and vessel junction formation. Quantitative analysis (Fig. 1i) showed that loop formation increased to approximately 145 ± 2% of control levels (*p* < 0.05), and tube length rose to 190 ± 4% of control (*p* < 0.01), reflecting significant expansion of the endothelial network. The most substantial change was observed in tube width, which increased to approximately 380 ± 40% of control (*p* < 0.001), suggesting formation of broader vessel-like structures. Collectively, these findings highlight the robust pro-angiogenic effects of EXOs, which promote the development of longer, wider, and more highly interconnected endothelial tubes *in vitro*.

### 3.4. Printability of CS and CS–PANI–EXOs biomaterial inks

The printability and structural integrity of 3D-printed scaffolds were assessed for CS, CS–PANI with 250 μL PANI, CS–PANI with 500 μL PANI, and CS–PANI with 750 μL PANI biomaterial inks and the results are presented in Table 1. The printed scaffolds were evaluated based on their adherence and uniformity to the intended 3D structure infill pattern.

**Table 1:** Visual depictions and targeted 3D infill geometries of various 3D-printed scaffolds, including CS and CS–PANI

The desired 3D structure infill pattern	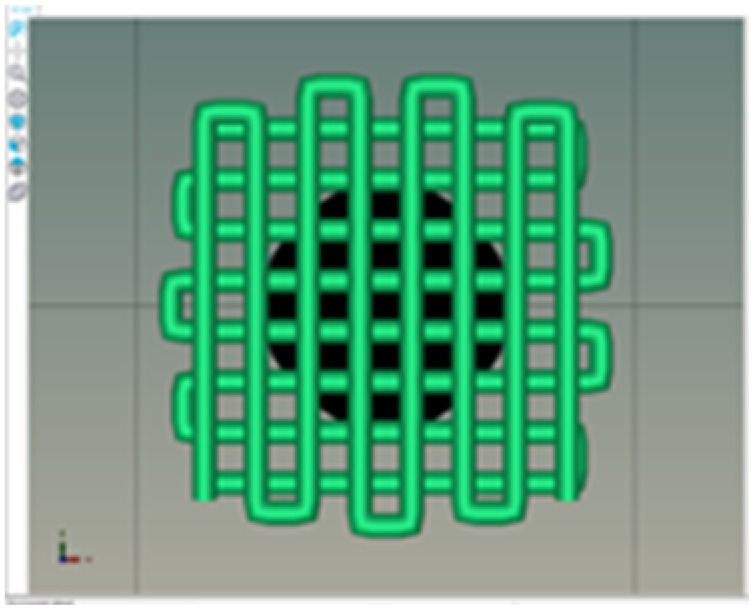			
Various 3D printed scaffolds	CS	CS–PANI (250 μL)	CS–PANI (500 μL)	CS–PANI (750 μL)
Images of 3D printed scaffolds	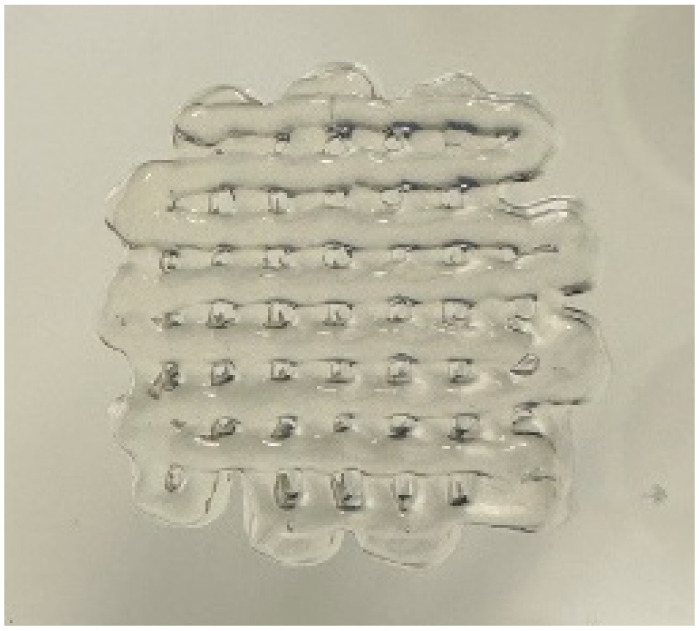	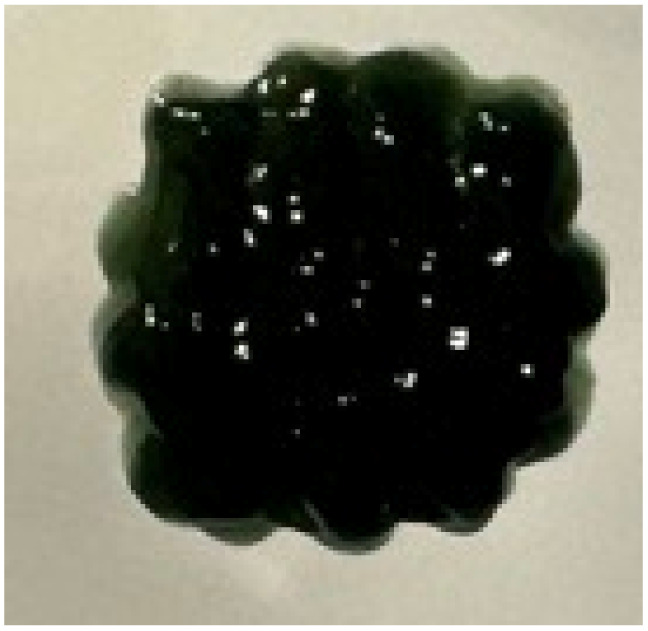	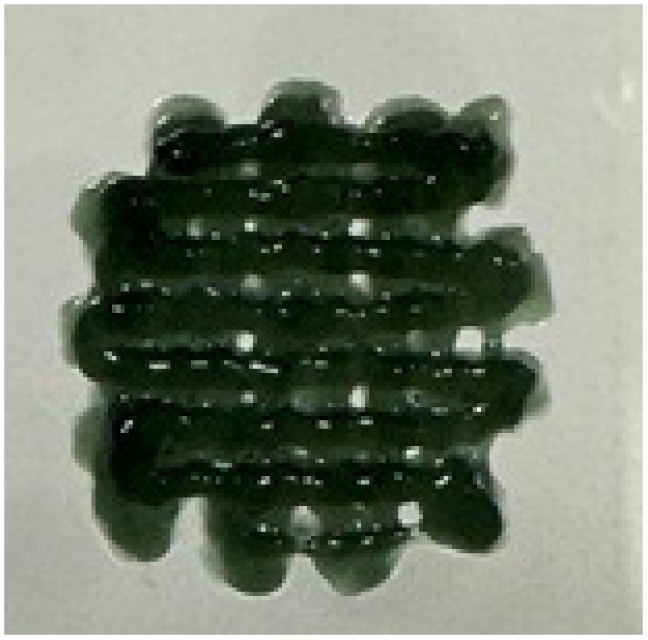	Failed to print due to high viscosity and nozzle blockage

The CS scaffolds, when printed without PANI, exhibited distinct and easily recognizable structures. Nevertheless, due to the absence of conductive components, these scaffolds have restrictions when it comes to applications that necessitate electrical properties. Although these scaffolds maintained their form (Table 1), they did not demonstrate the same level of durability as scaffolds containing PANI. The addition of 250 μL of PANI altered the visual appearance and structural integrity of the scaffold. The printed scaffold exhibited reduced clarity and a partially compressed appearance in comparison to the intended 3D configuration. The inadequate concentration of PANI resulted in low viscosity, which in turn led to poor shape retention and insufficient print resolution. The formulation exhibited a dilute consistency and lacked the requisite mechanical robustness to maintain the desired structure. The scaffolds containing 500 μL of PANI exhibited superior printability and structural integrity. As it demonstrated in the Table 1, these scaffolds closely resemble the intended 3D structure infill pattern, exhibiting distinct lines and stable forms. This concentration achieved an ideal equilibrium, leading to the desired thickness for easy extrusion and satisfactory mechanical properties to preserve the printed form. This formulation effectively attained the desired infill pattern, showcasing exceptional printability compared to the other tested formulations. The addition of 750 μL of PANI resulted in a high viscosity of the ink, which posed significant difficulties during the printing procedure. The elevated viscosity impeded the smooth flow of material through the nozzle, resulting in blockage and an incapacity to generate uniform 3D structures. As a result, this concentration did not lead to the formation of a suitable 3D structure. Therefore, given the poor printability of both the 250 μL and 750 μL PANI formulations, for all subsequent experiments CS–PANI or CS–PANI–EXOs hydrogels were prepared using the 500 μL PANI concentration. This formulation was selected due to its superior printability and mechanical properties, ensuring consistent performance across all experiments.

### 3.5. Characterization of biomaterial inks

**Fig. 2:**
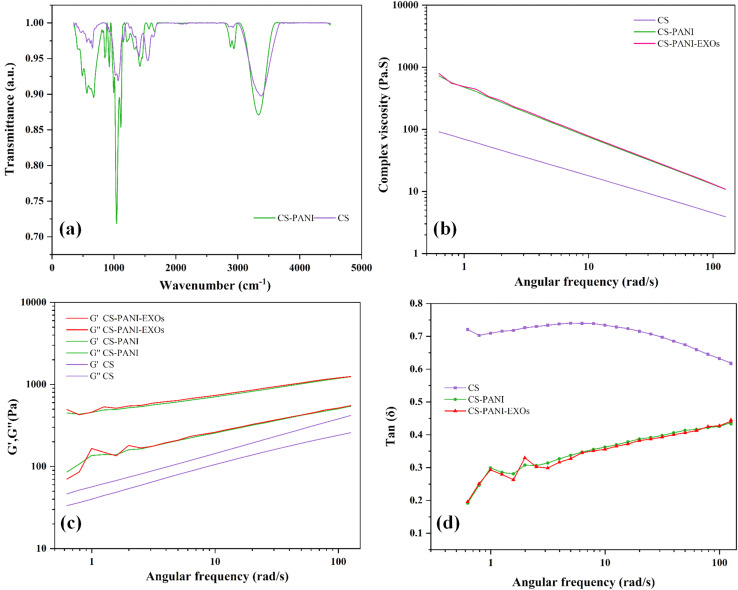
(a) FTIR spectra of CS and CS–PANI hydrogels. (b) Complex viscosity of CS and CS–PANI hydrogels as a function of angular frequency. (c) Storage modulus (*G*′) and loss modulus (*G*′′) of CS and CS–PANI hydrogels across a range of angular frequencies. (d) Tan(*δ*) *vs.* angular frequency of CS, CS–PANI, and CS–PANI–EXOs hydrogels.

The FTIR spectra revealed clear peaks that corresponded to specific functional groups in both hydrogels (Fig. 2a). CS hydrogel exhibited a prominent peak at 3380 cm^−1^, which indicated the presence of O–H stretching vibrations. This peak also encompassed the N–H vibrations that are characteristic of the amino groups in CS. The presence of amine groups was confirmed by the peak at 2927 cm^−1^, which corresponds to C–H stretching, and the peak at 1581 cm^−1^, which indicates N–H bending. The peaks observed at 1384 cm^−1^ and within the range of 1150–1021 cm^−1^ were associated with the vibrations of C–N bonds and the antisymmetric stretching of C–O–C and C–O groups, respectively.^29^ The CS–PANI hydrogel exhibited comparable peaks, along with supplementary signals that are distinctive of PANI. The prominent peak at 3294 cm^−1^ in the CS–PANI hydrogel suggests the existence of O–H and N–H functional groups, which aligns with the presence of both CS and PANI. The presence of amide groups, likely resulting from interactions between CS and PANI, was indicated by peaks observed at wavelengths between 1644–1542 cm^−1^. The presence of CH_3_ groups and the unique fingerprint of PANI in the composite hydrogel were confirmed by additional peaks observed at 1417 cm^−1^, 1263 cm^−1^, and 850 cm^−1^.^30,31^ Notably, the broadening and slight shift of the –OH/NH stretching peak from 3380 cm^−1^ (in CS) to 3327 cm^−1^ (in CS–PANI) suggests hydrogen bonding interactions between the amino groups of CS and the imine groups of PANI.^32^ These spectral differences, together with the appearance of characteristic PANI peaks (*e.g.*, at 1263 and 850 cm^−1^), may support the incorporation of PANI into the CS matrix and potential physical interactions within the composite hydrogel.

The rheological properties of the hydrogels were evaluated by measuring the complex viscosity, storage modulus (*G*′), and loss modulus (*G*′′) at different angular frequencies and the results are shown in Fig. 2b and c. The CS–PANI and CS–PANI–EXOs hydrogel consistently exhibited greater complex viscosity compared to the CS hydrogel, suggesting a stronger network structure attributed to the presence of PANI (Fig. 2b). The *G*′, which represents the elastic behavior, was higher than the *G*′′, indicating that all hydrogels primarily displayed solid-like properties. Significantly, the *G*′ values were notably greater in the CS–PANI and CS–PANI–EXOs hydrogels in comparison to the CS hydrogel (Fig. 2c). Additionally, the tan(*δ*) values (Fig. 2d) show that the CS hydrogel maintains higher values across the frequency range, which indicates more viscous behavior. On the other hand, the CS–PANI and CS–PANI–EXOs hydrogels exhibit lower tan(*δ*) values, which signifies a more elastic or solid-like behavior. Furthermore, these results indicate no significant difference between the rheological properties of the CS–PANI and CS–PANI–EXOs hydrogels, demonstrating that the inclusion of EXOs did not affect the scaffold's viscosity or elastic behavior.

**Fig. 3:**
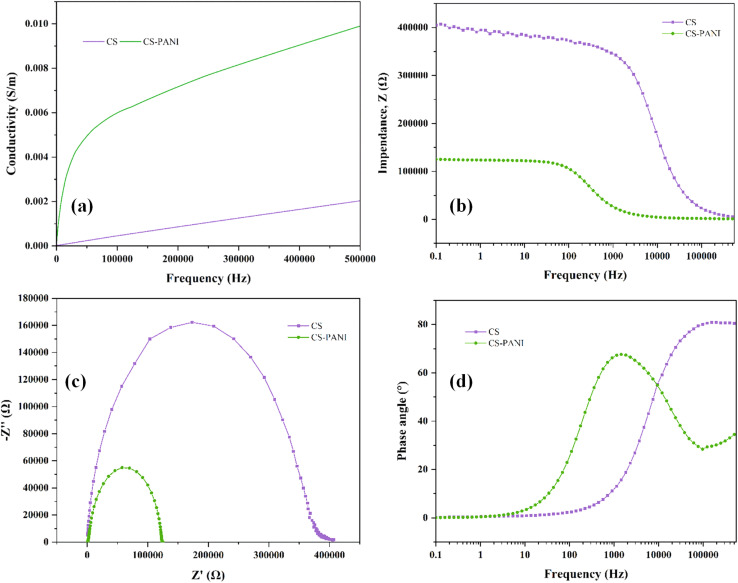
Electrochemical impedance spectroscopy (EIS) analysis of CS and CS–PANI hydrogels. (a) Conductivity spectra of CS and CS–PANI hydrogels measured across a frequency range from 1 Hz to 500 kHz. (b) Impedance *vs.* frequency plot demonstrating lower impedance in the CS–PANI hydrogel. (c) Nyquist plot (*Z*′ *vs. Z*′′) revealing a smaller semicircle for CS–PANI hydrogel, confirming higher conductivity. (d) Phase angle *vs.* frequency plot, highlighting the distinct phase behavior between the CS and CS–PANI hydrogels, with CS–PANI showing a lower phase angle indicative of improved conductive properties.

To assess the electrical conductivity of CS–PANI biomaterial ink, we conducted EIS, selecting 1 Hz as a key frequency for analysis. This is based on the understanding that at low frequencies such as 1 Hz, resistive currents dominate, allowing for a more accurate assessment of a biomaterial's conductivity relevant to its interaction with electroactive tissues.^33^ The impedance spectra for both hydrogels, measured across a frequency range spanning from 1 Hz to 500 kHz, are displayed in Fig. 3a. The result indicates that the CS–PANI hydrogel consistently exhibited significantly higher conductivity across all frequencies in comparison to the CS hydrogel. At a frequency of 1 Hz, the conductivity for CS–PANI was approximately 0.002 S m^−1^, whereas for CS it was significantly lower at around 0.0005 S m^−1^. The higher conductivity in CS–PANI remained consistent across the tested entire frequency range. The impedance (*Z*) values as a function of frequency are illustrated in Fig. 3b. The CS–PANI hydrogel demonstrated markedly reduced impedance compared to the CS hydrogel at all frequencies, thereby affirming its enhanced conductive characteristics. The Nyquist plot (Fig. 3c) illustrates the correlation between *Z*′ (real part) and *Z*′′ (imaginary part) for both hydrogels. The CS–PANI hydrogel exhibited a significantly smaller semicircle than the CS hydrogel, signifying reduced charge transfer resistance and enhanced electrical conductivity. Finally, the phase angle as a function of frequency is illustrated in Fig. 3d. CS–PANI exhibited a reduced phase angle at elevated frequencies relative to CS, indicating a more resistive and conductive nature in the CS–PANI hydrogel, thereby making it more appropriate for applications necessitating improved electrical communication, such as cardiac tissue engineering. Moreover, based on our EIS data and the impedance at 0.1 Hz, the calculated DC conductivity of the CS–PANI hydrogel was approximately 7.54 × 10^−3^ S cm^−1^. This value is consistent with previously reported conductivities for cardiac tissue scaffolds and is sufficient to support electrical signaling between cardiomyocytes.^34,35^

### 3.6. Distribution and release kinetics of EXOs

**Fig. 4:**
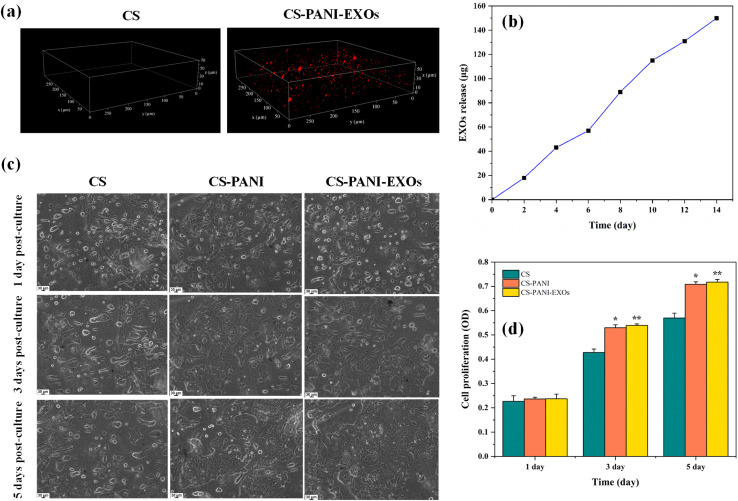
(a) 3D confocal images showing the distribution of EXOs within CS and CS–PANI–EXOs hydrogels. The red fluorescence indicates the presence of EXOs, confirming successful incorporation in the CS–PANI–EXOs hydrogel. (b) Release profile of EXOs from the CS–PANI–EXOs hydrogel over 14 days, demonstrating a gradual and controlled release. (c) Phase-contrast microscopic images showing the morphology and distribution of HL-1 cells on CS, CS–PANI, and CS–PANI–EXOs scaffolds at 1, 3, and 5 days post-culture. (d) Quantitative analysis of cell proliferation on different scaffolds measured by OD at 570 nm at 1, 3, and 5 days post-culture. The data reveal significantly higher cell proliferation on CS–PANI (*p < 0.05) and CS–PANI–EXOs (**p < 0.01) scaffolds compared to CS scaffolds.

The 3D confocal images revealed the distinct existence of EXOs within the hydrogel, indicated by intense red fluorescence evenly distributed throughout CS–PANI biomaterial ink (Fig. 4a). In contrast, CS hydrogel used as a control did not contain EXOs and did not exhibit fluorescence. It should be noted that the EXO fluorescence image is qualitative and limited by optical scattering in the hydrogel, the red signal appears somewhat diffuse. Nonetheless, the presence of red fluorescence throughout the scaffold indicates uniform EXO incorporation. The EXOs release from the CS–PANI–EXOs hydrogel was tracked for a duration of 14 days using dynamic dialysis. The Fig. 4b illustrates the release profile, which demonstrates a gradual pattern of release. The release starts off slowly and then gradually increases, ultimately reaching approximately 150 μg of EXOs released by day 14. The consistent and controlled release of the CS–PANI–EXOs hydrogel indicates its efficacy for applications requiring prolonged therapeutic delivery.

### 3.7. Cell viability

To evaluate the biocompatibility and ability to promote cell growth of the 3D-printed scaffolds, HL-1 cells were cultivated on three different types of scaffolds: CS, CS–PANI, and CS–PANI–EXOs. The viability and proliferation of the cells were assessed using an MTT assay at 1, 3, and 5 days after they were initially seeded. Fig. 4c displays phase-contrast microscopic images illustrating the morphology and distribution of HL-1 cells on the various scaffolds. On the first day, cells were sparsely distributed across all types of scaffolds. On the third day, there was a clear rise in the number of cells, particularly in the CS–PANI and CS–PANI–EXOs scaffolds, indicating significant growth. On the fifth day, the CS–PANI–EXOs and CS–PANI scaffolds showed the highest level of cell coverage, suggesting that it had better cell proliferation compared to the CS.

The Fig. 4d presented displays the quantitative analysis of cell proliferation, as determined by measuring the OD at a wavelength of 570 nm. There were no notable variations in cell viability among the three types of scaffolds on the first day. On the third day, CS–PANI and CS–PANI–EXOs scaffolds exhibited a notable rise in OD values in comparison to the CS scaffold indicating enhanced cell proliferation. On day 5, the trend persisted while CS–PANI–EXOs scaffolds continued to show high OD values, the difference between CS–PANI and CS–PANI–EXOs was not pronounced, with both outperforming the CS scaffold.

### 3.8. Beating of HL-1 and calcium transient analysis

**Fig. 5:**
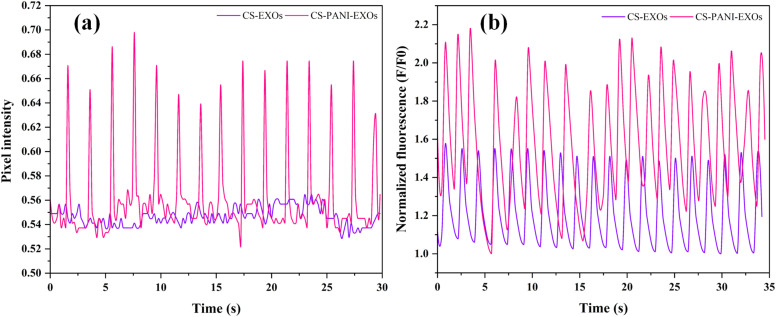
(a) Pixel intensity over time for HL-1 cells on CS–EXOs and CS–PANI–EXOs scaffolds, showing more pronounced peaks for the CS–PANI–EXOs scaffolds, indicative of rhythmic beating. (b) Normalized fluorescence (*F*/*F*_0_) over time for HL-1 cells on CS–EXOs and CS–PANI–EXOs scaffolds.

The rhythmic contraction, or beating behavior, of HL-1 cells on both conductive and non-conductive scaffolds was assessed using a phase-contrast microscope. Systematic observations were conducted in multiple regions of the scaffolds, covering both peripheral and central areas, in order to ensure a comprehensive analysis. The cells' dynamic behavior was recorded through video recordings. The analysis unveiled clear disparities among the scaffold types. The HL-1 cells exhibited consistent beating throughout the entire conductive CS–PANI scaffolds (Video 1†), including the edges and center, with regular contractions. Conversely, the HL-1 cells cultured on the CS scaffolds that lacked conductivity did not exhibit any visible beating activity (Video 2†). The pixel intensity over time plot as shown in Fig. 5a exhibits clear peaks corresponding to the beating activity, which signifies consistent and rhythmic contractions. In contrast, the HL-1 cells grown on the non-conductive CS–EXOs scaffolds (control) did not show any noticeable rhythmic contractions, as indicated by the lack of significant changes in intensity over time. Calcium transients in HL-1 cells cultured on the various scaffolds, which are indicative of cellular excitability and signaling also were examined. The Image sequences from confocal microscope showed that cells on the conductive CS–PANI scaffolds exhibited more pronounced calcium fluxes in comparison to cells on the non-conductive CS scaffolds. More precisely, the cells on the CS–PANI–EXOs scaffolds (Video 3†) displayed intense calcium signals, suggesting a high level of cellular excitability and effective calcium regulation. On the other hand, the calcium transients observed in HL-1 cells on the CS–EXOs scaffolds (Video 4†) exhibited a notable reduction in intensity, characterized by slower and less intense signals. The decrease in activity is associated with the absence of beating behavior observed in these cells, which highlights the crucial role of scaffold conductivity in preserving the physiological functions of cardiomyocytes. The normalized fluorescence data (*F*/*F*_0_) over time, as shown in Fig. 5b, further emphasize these differences, with the CS–PANI–EXOs scaffolds showing significantly higher and more consistent calcium transient peaks compared to the CS–EXOs scaffolds.

### 3.9. Cx43 expression and localization in HL-1 cells on different scaffolds

**Fig. 6:**
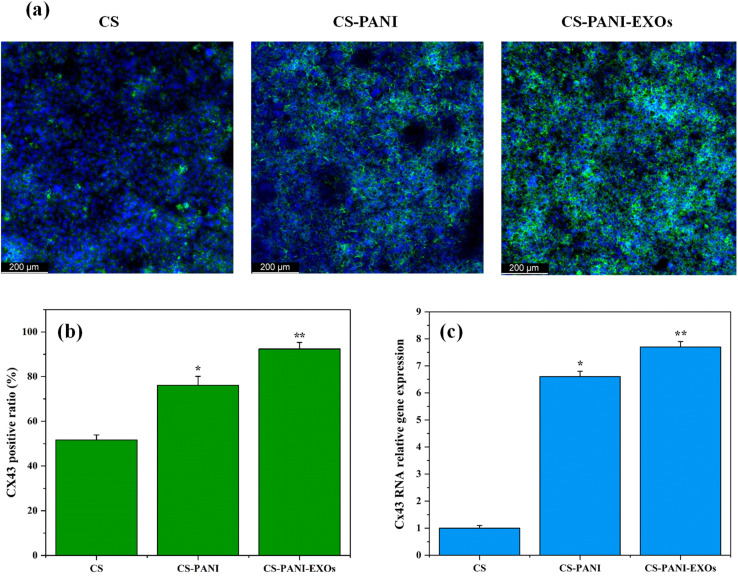
(a) Confocal images showing Cx43 expression (green) and nuclei (blue) in HL-1 cells cultured on CS, CS–PANI, and CS–PANI–EXOs scaffolds after 10 days. Cells on CS scaffolds exhibit limited and faint Cx43 staining, while cells on CS–PANI and CS–PANI–EXOs scaffolds show significantly elevated and more pronounced Cx43 expression, with the highest levels observed in the CS–PANI–EXOs scaffolds. (b) Quantitative analysis of the percentage of Cx43-positive cells, indicating significantly higher positive ratios in CS–PANI (76.08%) and CS–PANI–EXOs (92.37%) scaffolds compared to CS (51.64%) scaffolds (*p < 0.05, **p < 0.01, *n* = 4 films per condition). (c) Quantitative analysis of Cx43 RNA relative gene expression, showing significantly higher expression levels in cells cultured on CS–PANI and CS–PANI–EXOs scaffolds compared to CS scaffolds (*p < 0.05, **p < 0.01).

Immunocytochemistry was performed 10 days after seeding to evaluate the expression and location of the gap junction protein Cx43 in HL-1 cells cultured on different biomaterial inks hydrogels and the results are presented in Fig. 6a. The confocal images revealed clear variations in Cx43 expression between cells cultured on CS, CS–PANI, and CS–PANI–EXOs hydrogels. It demonstrated that cells on CS hydrogels exhibited limited and faint Cx43 staining, indicating a low level of expression of this gap junction protein. Conversely, cells on CS–PANI hydrogels displayed notably elevated levels of Cx43 expression, characterized by more pronounced and extensive staining. In the CS–PANI–EXOs hydrogels, the cells exhibited the most prominent Cx43 expression, characterized by distinct punctate staining patterns, indicating the presence of well-developed gap junctions. The quantitative analysis of Cx43 expression, as indicated by Fig. 6b revealed that the percentage of cells positive for Cx43 was significantly greater in CS–PANI hydrogels (76.08%) and CS–PANI–EXOs hydrogels (92.37%) compared to CS hydrogels (51.64%). This visually depicted the disparity, highlighting the notable augmentation in Cx43 expression resulting from the inclusion of PANI and EXOs.

qRT-PCR analysis of Cx43 expression, as shown in Fig. 6c, confirmed the visual observations. The expression of Cx43 was markedly greater in cells cultured on CS–PANI scaffolds as compared to CS scaffolds. The CS–PANI–EXOs and CS–PANI scaffolds demonstrated significantly higher levels of Cx43 expression compared to CS.

### 3.10. Gene expression profiling of H/R treated HL-1 cells *via* qRT-PCR analysis

**Fig. 7:**
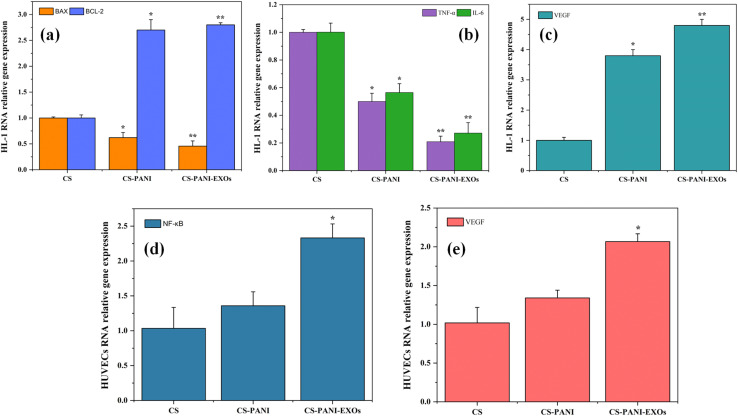
qRT-PCR analysis of HL-1 cells and HUVECs cultured on the hypoxic condition. (a) Comparative levels of expression of BAX and BCL-2 genes in HL-1 cells. (b) Expression levels of inflammatory cytokines TNF-α and IL-6 in HL-1 cells. (c) Expression levels of VEGF in HL-1 cells. (d) and (e) Expression level of NF-κB and VEGF genes respectively in HUVECs. Data are standardized based on the control group (CS scaffold) and expressed as mean ± SD. Statistical significance is indicated as *p < 0.05, **p < 0.01 compared to CA.

The impact of H/R on gene expression associated with apoptosis, inflammation, and angiogenesis in HL-1 cells cultured on different scaffolds was analyzed using qRT-PCR and the results are shown in Fig. 7 a–c. The relative expression levels of the genes BAX, BCL-2, TNF-α, IL-6, and VEGF were measured with GAPDH serving as the housekeeping gene. The data were standardized based on the expression levels in the control group (CS scaffold). Fig. 7a displays the comparative levels of expression of the pro-apoptotic gene BAX and the anti-apoptotic gene BCL-2. The expression levels of BAX and BCL-2 were similar on CS scaffolds. Nevertheless, the CS–PANI and CS–PANI–EXOs scaffolds exhibited a notable rise in BCL-2 expression, suggesting an improved anti-apoptotic effect, while BAX levels remained low, indicating a more potent anti-apoptotic effect. The levels of the inflammatory cytokines TNF-α and IL-6 are shown in Fig. 7b. The levels of both TNF-α and IL-6 were significantly reduced in the CS–PANI and CS–PANI–EXOs scaffolds compared to the CS scaffolds (*p < 0.05 and **p < 0.01, respectively). Furthermore, the CS–PANI–EXOs scaffold demonstrated a more prominent decrease in these cytokines, suggesting a more potent anti-inflammatory impact. Fig. 7c displays the levels of VEGF expression, which serves as a crucial indicator for angiogenesis. The CS–PANI–EXOs scaffolds exhibited a significant increase in VEGF expression in comparison to the CS–PANI and CS scaffolds.

In addition, the influence of the scaffolds on HUVECs under hypoxic conditions was evaluated by examining the levels of NF-κB and VEGF expression, as depicted in Fig. 7d and e, respectively. The CS–PANI–EXOs group exhibited significantly elevated expression of NF-κB, a critical mediator in the regulation of inflammatory responses, compared to the CS and CS–PANI groups (*p < 0.05). Furthermore, in relation to angiogenesis, the expression of VEGF was significantly increased in the CS–PANI–EXOs scaffolds compared to the other groups (**p < 0.01).

## 4. Discussion

This study aimed to create a new scaffold that combines the structural and conductive properties of CS and PANI with the biological functionality of EXOs. The motivation arises from the need for effective strategies to regenerate myocardium after MI, addressing both electrical signal propagation and tissue vascularization. Our findings demonstrate that the integration of PANI and EXOs yields synergistic benefits *in vitro*: improved cardiomyocyte survival and connectivity under stress, alongside enhanced angiogenic activity of endothelial cells. These outcomes suggest that such an EXO-functionalized conductive scaffold could be a promising component of future cardiac repair therapies.

The DLS analysis indicated a relatively homogeneous isolated EXOs population. Consistency in size is essential for therapeutic purposes, as it guarantees consistent dosage and bioactivity.^36^ The efficient internalization of these EXOs by HL-1 cells also underscores their capacity to impact cellular reactions, affirming their applicability in regenerative medicine and investigations of cell signaling. The rheological analysis revealed that addition of PANI to the CS hydrogel greatly improved its complex viscosity and storage modulus, suggesting improved mechanical strength. These results are consistent with previous studies indicating that the incorporation of PANI enhances the storage modulus and mechanical strength of hydrogels.^37,38^ FTIR analysis showed that the CS hydrogel displayed distinctive peaks that confirmed the existence of O–H, N–H, C–H, and C–N groups, which are in line with the established chemical composition of CS. The presence of these functional groups may enhance the hydrogel's biocompatibility and structural properties.^39^ The incorporation of PANI into the CS–PANI hydrogel resulted in the emergence of new peaks, such as those corresponding to CH_3_ groups, in the FTIR spectra, confirming the successful integration of PANI into the CS matrix. In addition, EIS verified the improved electrical conductivity of the CS–PANI hydrogel, rendering it appropriate for applications that necessitate conductive biomaterials, such as cardiac tissue engineering.^40^ Previous studies have demonstrated that the inclusion of PANI in hydrogels enhances their electrical conductivity.^41,42^

A key feature of our system is the sustained release of EXOs. This offers long-lasting therapeutic benefits, which may enhance the restoration of injured myocardial tissue.^43^ Moreover, this approach addresses the drawbacks of directly transplanting stem cells, such as low cell retention and survival rates, by harnessing the paracrine signaling abilities of EXOs. The EXOs-mediated biological effects observed *in vitro* were significant. EXOs markedly enhanced endothelial cell migration (scratch assay) and capillary tube formation. Angiogenesis is a complex process involving endothelial migration, alignment, and lumen formation, and it is crucial for reestablishing blood circulation to ischemic tissues and guaranteeing the supply of oxygen and nutrients required for tissue regeneration.^44^ HUVECs, which are primary cells responsible for the formation of blood vessels, are a highly suitable *in vitro* model for assessing the pro-angiogenic capabilities of therapeutic interventions.^45^ Our findings indicate that the inclusion of EXOs in the scaffold can enhance endothelial cell migration, which is a critical stage in angiogenesis. The significant increase in tube network metrics with EXOs indicates that factors carried by the EXOs (such as pro-angiogenic microRNAs or proteins) actively stimulate these processes. For instance, EXOs from MSCs are known to carry VEGF and other pro-angiogenic signals that can activate the AKT pathway in endothelial cells.^46^ Indeed, our HUVEC gene data showed increased VEGF expression when EXOs were present. This finding is significant because inadequate vascularization of engineered tissue constructs is a major hurdle in cardiac tissue engineering.

Our cell viability analyses implied that the primary improvement in the cell's growth may be attributed to the conductive characteristics of PANI, rather than the addition of EXOs. Although EXOs have the ability to modify cell behavior by transporting miRNAs and other bioactive molecules,^47–49^ their impact on cell proliferation in this specific scenario seemed to be restricted, emphasizing the need for further investigation. Previous studies have suggested that non-conductive hydrogels can impede cell communication, leading to reduced cardiomyocytes viability and function due to the absence of electrical coupling.^50^ By incorporating PANI into the scaffold, we enhanced the electrical conductivity. This improvement may not only facilitate the natural electromechanical functions of the cardiomyocytes but also enhance cell attachment, elongation, and maturation.^51–53^ The viability of HL-1 cells in our study was likely improved due to these factors, which align with the results of other studies on conductive hydrogels.^54^

The observation of rhythmic beats and elevated calcium transient signals (Fig. 4) in HL-1 cells on conductive scaffolds emphasizes the crucial significance of electrical conductivity in preserving cardiomyocyte functionality. The elevated calcium transients observed in HL-1 cells cultivated on conductive scaffolds suggest an efficient calcium regulation, which is essential for preserving the contractile function of the cardiac muscle.^55^ On the other hand, scaffolds that are not conductive do not give the required electrical signals, leading to less coordinated and weaker muscle contractions. This is supported by the decreased calcium activity and the lack of beating behavior in these cells. In addition, the conductive scaffolds enhance cell–cell communication *via* gap junctions which facilitate the direct exchange of ions and electrical signals between neighboring cells.^56,57^ This was further confirmed by the immunocytochemistry results which indicated improved cell–cell communication *via* gap junctions, evidenced by elevated Cx43 expression. Enhanced conductivity is crucial for preserving the electrical connection between cardiomyocytes, thus facilitating the development and operation of gap junctions. In addition, the inclusion of EXOs in the conductive scaffolds may enhance this effect. EXOs contain signaling molecules that may enhance the formation and maintenance of gap junctions, resulting in elevated levels of Cx43 expression.^58,59^

The qRT-PCR analysis demonstrated that the CS–PANI and CS–PANI–EXOs scaffolds effectively suppressed cell death, decreased inflammation, and stimulated angiogenesis in HL-1 cells after 24 h of hypoxic exposure. The upregulation of BCL-2 and downregulation of BAX suggest a diminished apoptotic response.^60–62^ The gene expression profile observed in this study suggests that the conductive CS–PANI scaffolds, whether with or without EXOs, establish a conducive environment that improves cell viability under H/R stress, likely by modulating apoptotic pathways. Conductive scaffolds have been shown to enhance cell survival under stress conditions, likely due to their ability to support cellular electrical activity and reduce apoptosis-related pathways, as evidenced in various tissue engineering studies.^23,63,64^ Reduced levels of inflammatory cytokines TNF-α and IL-6 suggest a notable decrease in inflammation, particularly in the CS–PANI–EXOs scaffold, which showed the most significant reduction.^65,66^ Previous studies have shown that the conductive characteristics of PANI help to stabilize the cellular environment, thereby reducing stress-induced inflammatory responses.^67,68^ In addition, research has confirmed that EVs, especially EXOs, have the ability to transport miRNAs and other biologically active substances that can block the NF-κB pathway, resulting in a decrease in the production of pro-inflammatory cytokines.^69–71^ The upregulated expression of VEGF in the CS–PANI and CS–PANI–EXOs scaffolds underscores their ability to stimulate angiogenesis, with the most significant increase observed in the presence of EXOs. VEGF plays a critical role in the formation of new blood vessels, which is necessary for the process of tissue repair and regeneration.^72^ EXOs may promote the growth of new blood vessels by transporting angiogenic factors and miRNAs that increase the expression of VEGF or activate pathways involved in angiogenesis.^73,74^ In HUVECs, the CS–PANI–EXOs scaffold markedly elevated NF-κB expression, which suggests that EXOs may play a role in modulating inflammatory pathways rather than merely increasing inflammation. This modulation is crucial for processes such as angiogenesis, as NF-κB activation is known to contribute to vascular repair mechanisms. Furthermore, the scaffold significantly enhanced VEGF expression, which indicates a strong pro-angiogenic effect. These findings are supported by studies demonstrating that EXOs can carry miRNAs like miR-92a-3p that promote angiogenesis by modulating target genes involved in endothelial function.^75–78^

Despite these promising *in vitro* results, several limitations and challenges need consideration for successful clinical translation. First, our study is restricted to cell lines and *in vitro* models. While the HL-1 cell line is a well-established model for studying cardiomyocyte behavior, it may not fully recapitulate the complexity and physiological characteristics of adult human cardiomyocytes. Utilizing primary cardiomyocytes or induced pluripotent stem cell-derived cardiomyocytes in future studies could verify whether the observed benefits are reproducible in more physiologically relevant settings. Moreover, although we demonstrated angiogenic effects on endothelial cells *in vitro*, confirming that these effects translate into effective blood vessel formation *in vivo* will be an essential next step. Another important consideration is the method of scaffold delivery. Injectable hydrogels are commonly preferred for minimally invasive cardiac administration, whereas our 3D-printed patch would necessitate surgical implantation.^79^ However, epicardial patch application remains a feasible and established clinical approach, potentially providing mechanical support to the ventricular wall and serving as a reservoir for sustained release of therapeutic agents.^80,81^ Thus, our scaffold could be similarly utilized as an epicardial patch during open-heart surgery or minimally invasive thoracoscopic procedures. Furthermore, although our findings revealed significant biological responses, detailed molecular analyses such as protein-level validations (*e.g.*, western blotting for angiogenic and inflammatory markers) were not included in this initial study. These analyses are planned for future research to enhance our understanding of the underlying molecular mechanisms. Additionally, comprehensive mechanical characterizations extending beyond rheological assessments, alongside detailed SEM-based microstructural analyses, are essential and will further elucidate the mechanical integrity and structural features of our scaffold.

Overall, our findings highlight that while the direct impact of EXOs on particular cellular processes, such as viability and survival, may have certain limitations, their role in modulating inflammation and enhancing angiogenesis was notably significant. Additionally, the conductive properties of PANI substantially improved the scaffold's overall functionality, particularly under hypoxic conditions, as evidenced by our qRT-PCR and functional data. Future research addressing the aforementioned limitations will substantially bolster the clinical relevance and potential applicability of our scaffold design.

## 5. Conclusion

This study created a unique 3D-printed scaffold using CS and PANI with the purpose of encapsulating BMSCs–EXOs for the regeneration of myocardial tissue after MI. By incorporating PANI, the electrical conductivity of the scaffold was enhanced, leading to improvements in structural integrity, and overall performance in hypoxic conditions. Although the impact of EXOs on particular cellular processes such as viability was minimal, their impact on inflammatory response and angiogenesis in both HL-1 and HUVECs under hypoxic conditions was substantial. The collective impact of these effects indicates that the CS–PANI–EXOs scaffold has the potential to mitigate the limitations associated with the rapid clearance and degradation of EXOs *in vivo*, offering sustained therapeutic effects. However, additional *in vivo* investigations are required to validate its effectiveness, safety, and potential clinical utilization in the field of cardiac tissue engineering and regenerative medicine.

## Data availability

The data supporting the findings of this study are not publicly available due to [specific reasons, *e.g.*, intellectual property considerations or proprietary restrictions]. However, relevant portions of the data can be shared upon reasonable request to the corresponding author, subject to approval by [*e.g.*, relevant authorities, legal agreements, or other requirements].

## Conflicts of interest

The authors declare that no known competing financial interests exist.

## Supplementary Material

RA-015-D5RA02940F-s001

RA-015-D5RA02940F-s002

RA-015-D5RA02940F-s003

RA-015-D5RA02940F-s004
